# Long noncoding RNA C17orf91 is a potential prognostic marker and functions as an oncogene in ovarian cancer

**DOI:** 10.1186/s13048-016-0258-3

**Published:** 2016-08-17

**Authors:** Jun Li, Hailin Yu, Meili Xi, Xin Lu

**Affiliations:** 1 Obstetrics and Gynecology Hospital, Fudan University, Shanghai, 200011 China; 2 Department of Obstetrics and Gynecology of Shanghai Medical College, Fudan University, Shanghai, 200032 China; 3 Shanghai Key Laboratory of Female Reproductive Endocrine Related Diseases, Shanghai, 200011 China; 4 Current address: Department of Gynecology, Obstetrics and Gynecology Hospital of Fudan University, No.419, Fangxie Road, Shanghai, 200011 China

**Keywords:** Ovarian cancer, Long noncoding RNA, C17orf91, MYC

## Abstract

**Background:**

This study was aimed to explore the role of long noncoding RNA C17orf91 and its potential mechanisms in ovarian cancer development.

**Results:**

To assess its role in ovarian cancer, microarray datasets (GSE14407, GSE30587, and GSE17260) in Gene Expression Omnibus (GEO) were utilized to assess the expression and clinical significance of C17orf91 in ovarian cancer. Next, loss-of-function studies were performed to establish the role of C17orf91 and the underlying mechanisms in ovarian cancer development. It was found that elevated expression of C17orf91 was observed in omental metastases when compared with matched primary ovarian tumors(GSE30587, *P* = 0.016). Moreover, Log Rank analysis revealed that increased expression of C17orf91 was associated with shorter progression free survival(PFS)(HR = 1.90(1.19-3.03), *P* = 0.008). Overall survival(OS) also showed a similar trend, but did not reach statistical significance(HR = 1.75(0.97-3.13), *P* = 0.061). Loss-of-function studies further demonstrated that C17orf91 repression impaired migration, invasion and viability of ovarian cancer cells, and downregulated the pro-metastatic gene, MYC, at both mRNA and protein level.

**Conclusion:**

Collectively, our findings revealed that C17orf91 was a potential prognostic marker and functioned as an oncogene in ovarian cancer. It remains to be seen whether modulation of C17orf91 expression will cause phenotypic changes in vivo.

**Electronic supplementary material:**

The online version of this article (doi:10.1186/s13048-016-0258-3) contains supplementary material, which is available to authorized users.

## Background

Ovarian cancer is the most lethal gynecological cancer and the majority of the patients are not diagnosed until advanced stages. Over the past decades, little improvement in overall survival has been achieved despite advances in chemotherapeutic agents [1]. Better understanding of the mechanisms involved in ovarian cancer development is of great importance in overcoming this malignancy.

Compelling evidence have indicated a large-scale regulatory network generated by noncoding RNAs, including microRNAs(miRNAs) and long noncoding RNAs(lncRNAs). Integrated analyses of the cancer genome and transcriptome have identified profound alterations in noncoding genes [2, 3]. Although numerous studies have helped unveiling the functions of miRNAs in various cancers, only a small number of functional lncRNAs have been thoroughly characterized to date. So far, lncRNAs have been involved in the regulation of various cellular processes, including but not limited to cell growth, cell cycle, apoptosis and motility [4]. C17orf91, also known as MIR22HG or MGC14376, is a long non-coding RNA(lncRNA) located on chromosome 17p13 [5, 6]. It is the host gene of miR-22 [5, 6]. Previous studies have linked miR-22 to a great number of activities, such as tumorigenesis, epigenetic modification, embryonic development [7]. However, the function of its host gene C17orf91 has not yet been defined in any conditions, including cancer [8, 9].

In this study, we tried to analyze C17orf91 expression and its clinical significance in ovarian cancer and to explore its role in ovarian cancer development.

## Methods

### GEO datasets

The publicly available microarray datasets used in our study is described in GEO database(GSE14407, GSE30587 and GSE17260). GSE14407 dataset was used to explore the differential expression of C17orf91 (probe ID: 214696_at) between normal ovarian surface epithelium(*n* = 12) and primary ovarian cancers(*n* = 12). GSE30587 dataset was used to determine the differential expression of C17orf91 (probe ID: 8011193) between paired primary ovarian cancer tissues(*n* = 9) and their corresponding omental metastases(*n* = 9). Relationship between C17orf91 expression (probe ID: A_23_P49610) and ovarian cancer outcome was determined using the gene expression data in 110 ovarian cancer patients(GSE17260).

### Cell lines

Human serous ovarian cancer cell Hey was originally obtained from M.D. Anderson Cancer Center (Houston, TX) and cultured in RPMI medium 1640 plus 10 % fetal bovine serum with penicillin/streptomycin. 293 T cell line was originally obtained from the cell bank of Chinese Academy of Science (Shanghai) and cultured in DMEM plus 10 % fetal bovine serum with penicillin/streptomycin.

### Constructs

PLKO-Scramble-shRNA was purchased from Addgene(Addgene #1864). PLKO-C17orf91-shRNA plasmids were constructed according to PLKO.1 protocol. The sequences of C17orf91 shRNA were obtained from The RNAi Consortium (TRC, MISSION® TRC shRNA library, Sigma) and shown in Additional file 1: Table S1.

### Lentiviral infection

For producing lentiviral particles, 293 T cells were seeded in 3.5-cm dishes at a density of 4 × 10^5^ per dish and transfected with PLKO-Scramble-shRNA/PLKO-C17orf91-shRNA, as well as packing plasmids psPAX2 (0.75 μg) (Addgene#12260) and pMD2.G (0.25 μg) (Addgene #12259) using Attractene Transfection Reagent (QIAGEN). Forty-eight hours post transfection, virus-containing medium (2 ml) was harvested, filtered, mixed with 2 ml of freshsly prepared medium, supplemented with 8 μg/ml polybrene (Sigma) and added to 3 × 10^5^ Hey cells seeded in a 3.5-cm dish the day before. Puromycin (2 μg/ml) was supplemented 48 h after infection. The cells were selected for 2 days and then used for various assays.

### QPCR

Briefly, reverse transcription reactions were carried out using RevertAid^TM^ First Strand cDNA Synthesis Kit (Fermentas). QPCR was performed using SYBR® *Premix Ex Taq*™ II (Perfect Real Time) Kit (TaKaRa, Dalian, China) in ABI PRISM 7500 Sequence Detection System (Applied Biosystems). Quantitative analysis was performed using Comparative CT method. The relative expression of each gene was normalized to the expression of GAPDH. Primers used in present study were shown in Additional file 1: Table S1.

### Western blot assay

Whole cell extracts were prepared in chilled RIPA lysis buffer(Beyotime, China). 30 μg of lysates protein were separated by SDS-PAGE using a 10 % polyacrylamide gel and transferred to 0.45 μm PVDF membrane(Millipore). Membranes were blocked with 5 % non-fat milk in PBS containing 0.05 % Tween-20, blotted with MYC antibody(1:1000, CST) or GAPDH antibody(1:3000, beyotime) overnight, followed by goat anti-mouse antibody. The images were scanned by LAS4000 device.

### Measurement of cell migration and invasion

RTCA CIM-Plate was used to explore the effect of C17orf91 on migration and invasion of ovarian cancer cells. Migration and invasion assays were performed according to protocols supplied by the manufacturers. For invasion assay, the inserts were coated with BD Matrigel (B.D. Biosciences). Briefly, 4 × 10^4^ Scramble-shRNA- and C17orf91-shRNA-transfected Hey cells were added into the RTCA CIM-Plate and monitored by xCELLigence (Roche) for 36 h.

### Measurement of cell viability

RTCA E-plate was used to explore the effect of C17orf91 on cell viability. Briefly, 2 × 10^3^ Scramble-shRNA- and C17orf91-shRNA-transfected Hey cells were added into the RTCA E-Plate and monitored by xCELLigence (Roche) for 96 h.

### Statistical analysis

All data were analyzed with SPSS statistic software (SPSS 16.0). Student’s *t* test (two-tailed) was used to compare two groups. The Log-rank test was used to determine the relationship between C17orf91 expression and clinical outcomes(progression free survival and overall survival). The Kaplan-Meier method was used to generate survival curves. *P*-value <0.05 was considered statistically significant.

## Results

### Differential expression of C17orf91 between OSE, primary tumors and omental metastases

**Fig. 1:**
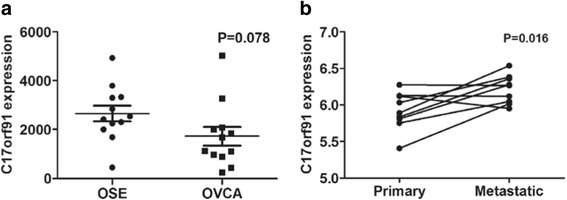
Differential expression of C17orf91 between OSE, primary tumors and omental metastases. **a** C17orf91 expression appeared to be decreased in primary tumors compared with OSE, but do not reach statistical significance(GSE14407). **b** Paired *t*-test shows that C17orf91 expression was significantly elevated in omental metastases relative to corresponding primary tumors(GSE30587)

To explore the clinical significance of C17orf91 in ovarian cancer development, we first determined the differential C17orf91 expression between serous ovarian cancer tissues and normal ovarian surface epithelium(OSE) with resort to the GEO datasets. It was found that C17orf91 expression appeared to be downrelulated in ovarian cancer tissues compared with OSE, though without statistical significance(GSE14407, Fig. 1a, *P* = 0.078). However, elevated expression of C17orf91 was observed in omental metastases when compared with matched primary ovarian tumors(GSE30587, Fig. 1b, *P* = 0.016).

### The prognostic value of C17orf91 expression in ovarian cancer

**Fig. 2:**
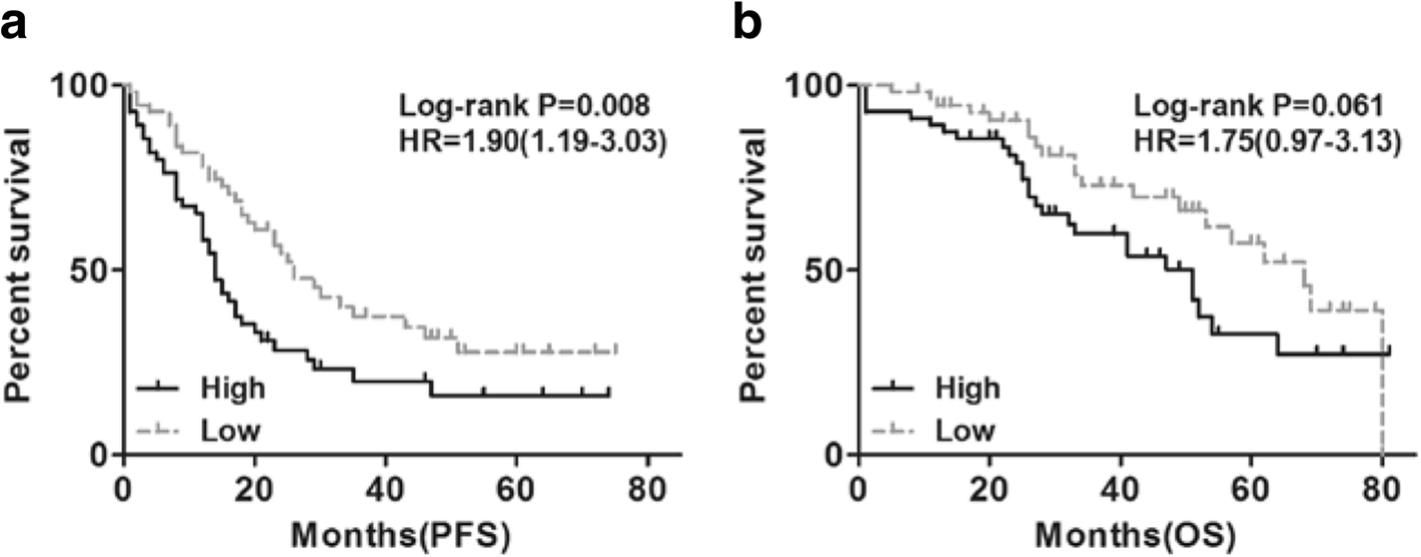
The prognostic value of C17orf91 expression in ovarian cancer(GSE17260). **a** Log Rank (Mantel-Cox) analysis revealed that increased expression of C17orf91 was associated with shorter progression free survival(PFS). **b** Overall survival(OS) also showed a similar trend, but did not reach statistical significance. “Low” and “High” were classified according to the C17orf91 expression level. The median expression value for C17orf91 was used as the cutoff point

Next, we assessed the prognostic value of C17orf91 expression by taking advantage of the microarray dataset (GSE17260) in Gene Expression Omnibus. Interestingly, Log Rank (Mantel-Cox) analysis revealed that increased expression of C17orf91 was associated with shorter progression free survival(PFS) (Fig. 2a, HR = 1.90(1.19-3.03), *P* = 0.008). Overall survival(OS) also showed a similar trend, but did not reach statistical significance(Fig. 2b, HR = 1.75(0.97-3.13), *P* = 0.061).

### Oncogenic role of C17orf91 in ovarian cancer

**Fig. 3:**
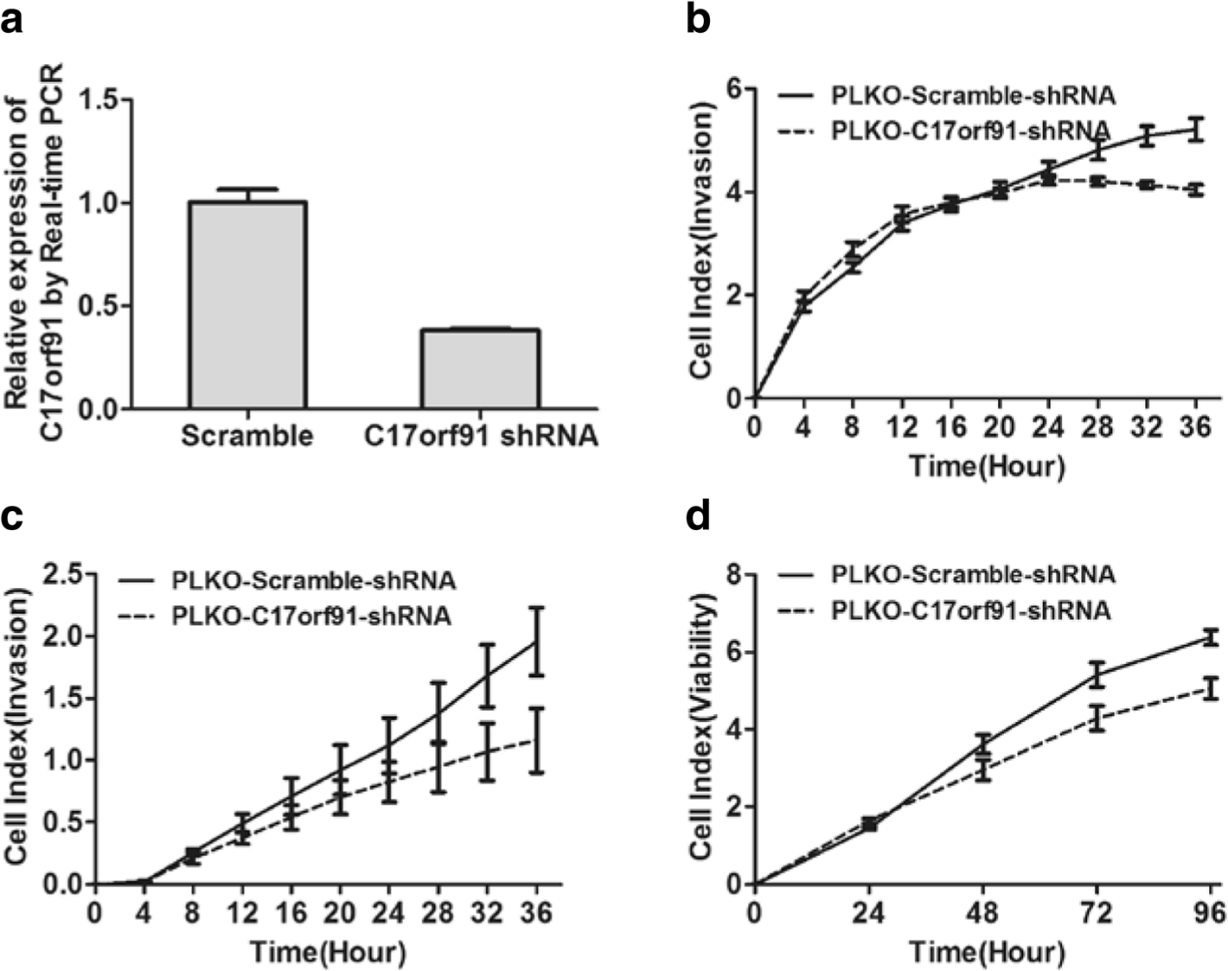
The oncogenic role of C17orf91 in ovarian cancer monitored by xCELLigence. **a** Construction of Hey cells that stably inhibited C17orf91 expression. **b, c, d** Inhibition of C17orf91 repressed migration (**b**), invasion (**c**), and viability (**d**) of ovarian cancer cells

To explore the potential role of C17orf91 in ovarian cancer, we performed loss-of-function studies in Hey cells. Stable C17orf91-shRNA clones as well as their respective control clones were generated and examined for C17orf91 mRNA by Real-time RT-PCR (Fig. 3a). Interestingly, C17orf91 inhibition significantly decreased migration, invasion and viability of Hey cells (Fig. 3b–3d).

### C17orf91 reglulated MYC expression in ovarian cancer

**Fig. 4:**
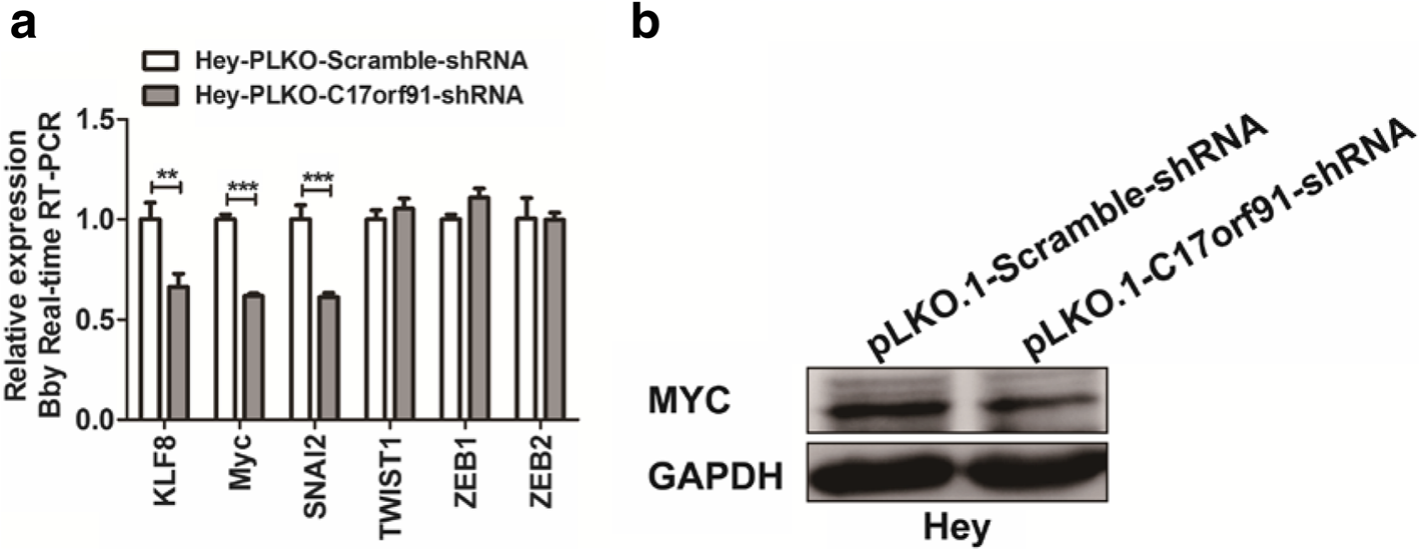
The regulatory effects of C17orf91 on the expression of pro-metastatic genes. **a** Knockdown of C17orf91 inhibited KLF8, MYC and SNAI2 mRNA expression in ovarian cancer cells. **b** C17orf91 downregulation decreased the protein level of MYC

To further investigate the mechanism by which C17orf91 elicited its oncogenic role, we explored whether C17orf91 could regulate key pro-metastatic genes, such as KLF8 [10], MYC [11], SNAI2 [12], TWIST1 [13], ZEB1 [14] and ZEB2 [15] in ovarian cancer. It was found that C17orf91 repression could reduce the expression of KLF8, MYC and SNAI2 at mRNA level (Fig. 4a). Subsequent western blot assay was focused on MYC partly because it was the most obviously altered gene. It was found that C17orf91 downregulation also decreased the protein level of MYC (Fig. 4b).

## Discussion

C17orf91, also known as MIR22HG, was a stress responsive lncNRA [16]. Previous studies showed that stress responsive lncRNAs might be linked to cancer progression [17–19]. Knockdown of LSINCT5 significantly impaired the proliferation of both breast and ovarian cancer cells [17]. Another stress responsive lncRNA which might also be involved in cancer progression is PRINS(psoriasis susceptibility-related RNA gene induced by stress). It was found that PRINS played a protective role in cells exposed to stress, and moreover, elevated PRINS expression in the epidermis might contribute to psoriasis susceptibility [18]. Further studies revealed that PRINS could regulate the expression of G1P3, an inferno-inducible gene with anti-apoptotic effects in cancer cells [19]. In this study, we also demonstrated that the stress responsive lncRNA C17orf91 could regulate the migration, invasion and viability of ovarian cancer cells.

Additionally, we characterized the clinical significances of C17orf91 in ovarian cancer tissues by taking advantages of publicly available microarray datasets in GEO database. It was noticeable that C17orf91 expression appeared to be downregulated in ovarian cancer tissues compared with OSE though without statistical significance. Indeed, such findings were not only limited in our study. For example, the well-known pro-metastatic gene miR-10b was found to be downregulated in primary breast tumors compared with normal breast tissues [20, 21]. Our findings, together with findings from other laboratories, have indicated that oncogenes are not always upregulated in cancer tissues when compared with corresponding normal tissues. This may also be applicable for tumor suppressors which are commonly downregulated in cancer tissues relative to normal controls.

Previous studies indicated that decreased C17orf91 expression was associated with a worse prognosis in breast and lung cancer patients [8, 9, 22]. However, our data revealed that increased C17orf91 expression correlated with shorter PFS and OS. These discrepant findings might be related to the fact that the prognostic value of C17orf91 was cancer specific. Mechanismly, we demonstrated that C17orf91 repression could downregulate the MYC expression at both mRNA and protein level, indicating that the oncogenic role elicited by C17orf91 might be partly mediated by the induction of MYC expression.

## Conclusion

In conclusion, our findings revealed that C17orf91 was a potential prognostic marker and functioned as an oncogene in ovarian cancer. It remains to be seen whether modulation of C17orf91 expression will cause phenotypic changes in vivo.

## Additional file

Additional file 1: Primers used in present study. (XLS 17 kb)
